# A longitudinal multi-centric cohort study assessing infant neurodevelopment delay among women with persistent postpartum depression in Nepal

**DOI:** 10.1186/s12916-024-03501-0

**Published:** 2024-07-08

**Authors:** Ashish KC, Jaya Chandna, Ankit Acharya, Rejina Gurung, Carin Andrew, Alkistis Skalkidou

**Affiliations:** 1 School of Public Health and Community Medicine, Sahlgrenska Academy, University of Gothenburg, Medicinargatan 18, Gothenburg, Sweden; 2 MARCH Center, London, School of Hygiene and Tropical Medicine , London, UK; 3 Research Division, Golden Community, Lalitpur, Nepal; 4 Department of Women’s and Children’s Health, Uppsala University, Uppsala, Sweden

**Keywords:** Infant’s delayed neurodevelopment, Postpartum depression, Infant young child development (IYCD) domain, Nepal

## Abstract

**Background:**

Infant neurodevelopment in the first years after birth is determined by multiple factors, including parental care and maternal mental wellbeing. In this study, we aim to assess the impact of persistent maternal depressive symptoms during the first 3 months postpartum on infant neurodevelopment at 6 months.

**Methods:**

Using a longitudinal cohort design, 1253 mother-infant pairs were followed up at 7, 45, and 90 days to assess postpartum depressive symptoms using the Edinburgh Postnatal Depression Scale (EPDS); infants were followed up at 6 months to assess neuro-developmental status using the WHO’s Infant and Young Child Development (IYCD) tool. A generalized linear regression model was used to assess the association between persistent postpartum depressive symptoms and infant neurodevelopmental delay at 6 months. A generalized linear mixed model (GLMM) with a hospital as a random intercept was used to assess the persistent postpartum depressive symptoms with an IYCD score. Linear regression was used to compare the IYCD scores between exposure groups.

**Results:**

In the study population, 7.5% of mothers had persistent depressive symptoms, and 7.5% of infants had neurodevelopmental delay. Infants born to mothers with persistent depressive symptoms had a higher proportion of neurodevelopmental delay than infants born to women without persistent symptoms (48.6% vs 5.1%; *p* < 0.001). In the adjusted regression model, infants whose mothers had persistent depressive symptoms at 7, 45, and 90 days had a 5.21-fold increased risk of neurodevelopmental delay (aRR, 5.21; 95% CI, 3.17, 8.55). Mean scores in the motor domain (12.7 vs 15.2; *p* < 0.001) and language domain (6.4 vs 8.5; *p* < 0.001) were significant when a mother had persistent depression vs. no depression. Mean scores in the general behavioral domain (5.9 vs 10.4, *p* < 0.001) and the socio-emotional domain (15.4 vs 17.7; *p* < 0.001) were significantly different when a mother had persistent depression vs no persistent depression.

**Conclusions:**

Our results suggest that 6-month-old infants are at higher risk for neurodevelopment delays if their mother reports persistent symptoms of depression from 7 to 90 days postpartum. The neurodevelopmental delay can be observed in all functional domains. Preventive intervention to reduce maternal postpartum depression may reduce the impact on infant developmental delay.

**Supplementary Information:**

The online version contains supplementary material available at 10.1186/s12916-024-03501-0.

## Background

Maternal depression is a global health problem, with an estimated 23.4 million mothers experiencing postpartum depressive symptoms each year and a prevalence rate of 17.2% [1]. Depressive episodes during the postpartum period range from sadness, changes in sleep and eating patterns, despair, crying spells, anxiety, irritability, feelings of isolation, mental liability, thoughts of hurting oneself and/or the infant, and even suicidal thoughts [1]. In South Asia, the rate of maternal depression is 22.2%, with the prevalence rate varying according to the timing and duration of breastfeeding, maternal education, income, life stress, gestational age at birth, and infant illness [1–4]. Infant and early childhood development is a maturational and interactive process that progressively develops perceptual, motor, cognitive, language, socio-emotional, and self-regulatory skills [5]. Parental psychopathology has been found to be a consistent and robust correlate of child maladjustment, particularly maternal depression [6–9].

More than 250 million children under the age of 5 were estimated to have developmental delays in LMICs (low- and middle-income countries) worldwide in 2015 [10], highlighting the need to improve access to multisectoral interventions that include health, nutrition, security and safety, responsive care, and early learning for parents [11]. Parents need support to provide nurturing care; they need learning materials and resources to support their children’s neurodevelopment [12]. The fundamental promotive childhood experiences come from the nurturing care and protection received from parents, family, and community, which have lifelong benefits [13].

There are consistent findings linking maternal depression to deficits in both socio-emotional and instrumental functioning. A landmark study conducted by the National Institute of Child Health and Early Development showed that children whose mothers reported depressive symptoms had poorer cognitive functioning and school readiness compared to children who mothers never reported depressive symptoms [14]. Although the literature provides both theoretical and limited empirical support for a reciprocal effects model between maternal depression and child development, a key question remains about the timing of these effects. Children whose mothers reported feeling depressed performed worse on measures of cognitive-linguistic functioning and were rated as less cooperative and more problematic [15, 16].

In Nepal, studies have shown that the population prevalence of maternal postpartum depressive symptoms ranges from 28 to 33% [17, 18], indicating that one-third of the women have depressive symptoms, which is higher than estimates (20% prevalence) made for mothers in LMIC [19]. Given the high burden of postpartum depressive symptoms in Nepal and the link between infant neurodevelopment and maternal mental health, this study aims to assess the impact of persistent maternal depressive symptoms during the first 3 months of the postpartum period on infant neurodevelopment at 6 months.

## Methods

### Design

This is a longitudinal cohort study design nested within an ongoing quality improvement study in 9 surveillance site hospitals in Nepal. From the women-infant pairs enrolled in the ongoing quality improvement study, a sample of women-infant pairs was selected and followed up until 6 months postpartum. The sample of women who delivered between April and July 2023 were assessed for depressive symptoms between April 28 and September 29, 2020, and developmental assessment follow-up at 6 months was conducted between October 10, 2020 and January 29, 2021 [20, 21].

### Study setting

The nine public referral hospitals included in the study represented the seven administrative provinces and the population of the country. Seti Provincial Hospital and Dadeldhura Hospital represented the marginalized population in the far western province; Surkhet Provincial Hospital catered service to the mid-western hilly population; Bheri, Lumbini, Janakpur, and Koshi Hospitals represented the population in the Southern plain province of the country; and Kaski Hospital represented the central hilly population of the country.

### Inclusion criteria

Mothers who had singleton live born infants were eligible for inclusion in the study and were approached for consent [22]. Mothers who experienced birth trauma and understood the Nepali language were also eligible to participate. Mothers who consented to the study were followed up at 7, 45, and 90 days for depressive symptoms using the Edinburgh Postpartum Depression Scale (EPDS), and infant neuro-developmental assessment was conducted at 6 months (180 days) using the World Health Organization’s Infant and Young Child Development (IYCD) tool.

### Sample

**Fig. 1:**
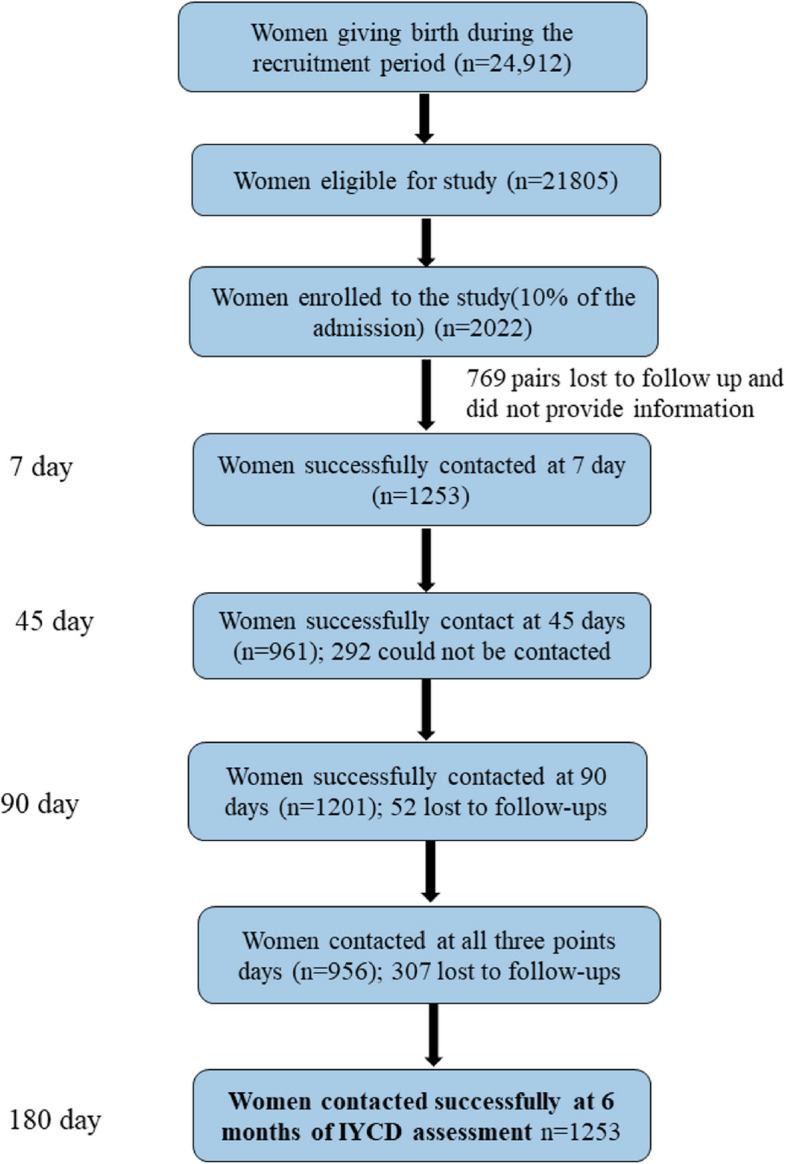
Study flow diagram

We randomly selected 10% of the mothers enrolled in the quality improvement study in the nine hospitals for this longitudinal cohort study using random sequence generation in an Excel spreadsheet with all participants. Of the 2022 mothers enrolled, longitudinal follow-up was conducted at 7, 45, 90, and 180 days (Fig. 1).

### Measure

#### Exposure

Symptoms of postpartum depression were measured using the EPDS. EPDS is a validated self-reported 10-question self-report questionnaire with a scale of 0 to 3 to measure depressive symptoms in postpartum mothers [23, 24], and for this study, a score of ≥ 12 was used to categorize mothers with depressive symptoms. Persistent depressive symptoms were defined as scores ≥ 12 at all three time points (7, 45, and 90 days).

#### Outcome

Infant neurodevelopment was measured using the WHO’s IYCD, an open-access, validated, mother-reported tool for child development in the domains of motor, language-cognitive, social-emotional, and general behavior [25, 26]. The IYCD was a 100-item parent report tool (40 fine motor and gross motor, 30 language, 20 socio-emotional, and 10 unscored behavioral items). Delayed infant neurodevelopment at 6 months was based on a centile score of 10 or less, based on previous literature [25, 26].

### Covariates

#### Ethnicity

In Nepal, the social class system determines the social access to resources. For the purpose of this study, ethnicity was divided into two social groups, first a disadvantaged social group which included janjati, muslim, madeshi, and dalit ethnic groups and an advantaged social group such as chettri-brahmin. Maternal education was categorized as uneducated (illiterate and unable to read and write) and educated (primary education and above).

#### Preterm birth

Based on the date of last menstrual cycle date, preterm birth was defined as birth occurring before 37 weeks of gestation; low birth weight was defined as birth weight < 2500 g, and infant sex was categorized as boy or girl.

#### Data collection

The hospital research surveillance team approached eligible women, informed them of the study, and enrolled those who provided written consent. The trained research surveillance team conducted telephone follow-ups at 7, 45, 90, and 180 days after delivery. During the telephone follow-up with the mother, sociodemographic, obstetric, postpartum depression (EPDS 10), and infant neurodevelopment (IYCD) data were collected using a semi-structured questionnaire from a tablet-based application.

#### Data management and analysis

**Fig. 2:**
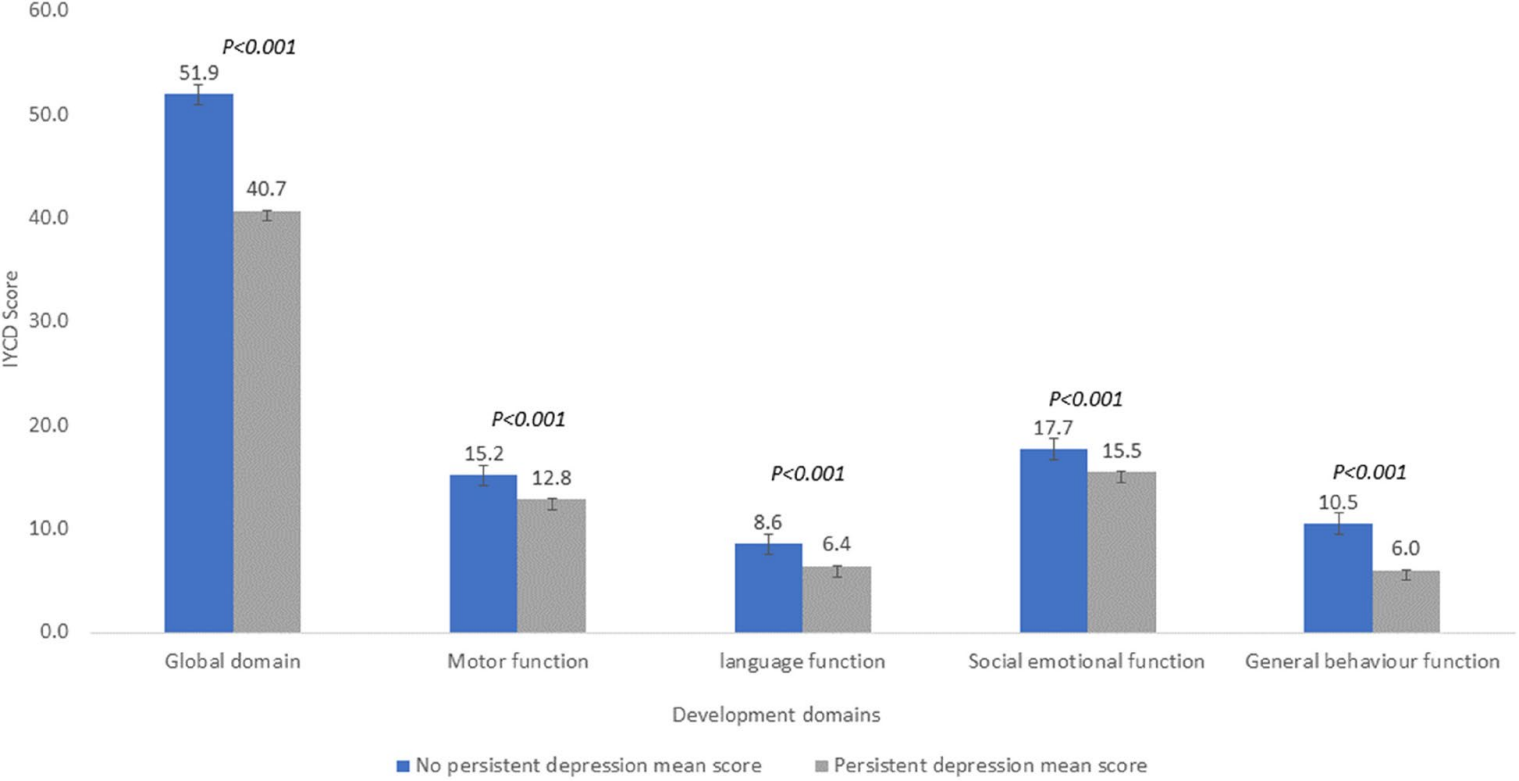
Scores in different functional domains among infants at 6 months born to mothers with/without depressive symptoms

Descriptive statistics on the prevalence of maternal depressive symptoms at 7, 45, and 90 days and persistent depressive symptoms were calculated in Additional file 1: Table S1. The IYCD as a score for a global developmental domain and subdomains was analyzed using percentile cut-offs calculated in Additional file 1: Table S2. Differences in the population characteristics (maternal ethnicity, maternal education), birth characteristics (preterm birth, LBW, and infant sex), maternal depression (exposure), and infants with developmental delay (outcome) were analyzed using Pearson’s chi-square, and Fisher’s exact test was done in Tables 2 and 3. We used three different statistical models to assess the causal relationship between maternal depression and infant neurodevelopment. In the *first statistical model*, a generalized linear regression model was used to assess the association between maternal depression at 7, 45, and 90 days and persistent postpartum depressive symptoms with an infant’s neurodevelopmental delay at 6 months, as shown in Additional file 1: Table S3. In the *second statistical model*, the generalized linear mixed model (GLMM) with the hospital as a random intercept was used. In this model, because there were not enough children with developmental problems in all the hospitals, we decided not to use a binary outcome and instead modeled the outcome as a continuous variable (IYCD6 continuous) using a mixed effects linear regression model with the hospital as a random intercept in Table 4. In the *third statistical model*, the mean score with standard deviation of IYCD and functional domain of infants whose mothers had persistent depression compared to infants whose mothers did not have persistent depression using linear regression model in Fig. 2 and Additional file 1: Table S2. The sociodemographic factors (ethnicity and maternal education) and biological factors (infant sex and prematurity) that differed in the exposure population were adjusted in all three statistical models. Statistical analysis was performed using IBM SPSS Statistics SPSS 28.0.2 version for generating supplementary tables and R program-4.3.3 version for generating main text tables and the “glmm” package for GLMM regression [27, 28]. Missing data for any exposure or outcome of interest were excluded at the pairwise level.

## Results

During the recruitment period, a total of 24,912 mothers delivered at the nine study hospitals. Of those who gave birth, 21,805 were eligible for enrollment, and 10% of them were randomly selected for this study. Of the 2022 mothers enrolled in this study, 1253 were successfully contacted during the 7 days follow-up to assess depressive symptoms, 961 were successfully followed up at 45 days (292 lost to follow-up), and 1201 were successfully followed up at 90 days (52 lost to follow-up). Of the mothers who were followed, 946 were successfully contacted at all three time points (307 were lost to follow-up at all three time points). At 6 months, 1253 mothers had a successful postnatal interview to assess the infant neurodevelopmental status using the IYCD reporting tool (Fig. 1).

### Maternal depression and population characteristics

Among the follow-up study cohort, the prevalence of mothers with depressive symptoms at 7, 45, and 90 days was 10.1% (*n* = 127), 11.6% (*n* = 111), and 9.6% (*n* = 115) respectively. The prevalence of mothers with persistent depressive symptoms was 7.5% (*n* = 71) (Additional file 1: Table S1). There was a statistical difference in the persistent depressive symptoms among mothers by social group (*p* < 0.001), maternal education (*p* < 0.001), and infant’s prematurity (*p* < 0.001) (Table 1).

**Table 1:** Sociodemographic and obstetric characteristics of mothers with or without persistent depressive symptoms (*n* = 946)

	Persistent depressive symptom		
	**No, *n* (%) **875 (92.5%)	**Yes, *n* (%) **71 (7.5%)	*p*-values
**Maternal ethnicity (social group)**			
Relatively advantaged^a^ (313)	309 (98.7%)	4 (1.3%)	< 0.001*
Relatively disadvantaged^b^ (633)	566 (89.4%)	67 (10.6%)	
**Maternal education**	0	1	
Educated (799)	761 (95.2%)	38 (4.8%)	< 0.001**
Not educated (101)	71 (70.3%)	30 (29.7%)	
**Sex of infant**			
Girl (390)	362 (92.8%)	28 (7.2%)	0.774**
Boy (555)	512 (92.3%)	43 (7.7%)	
**Preterm birth**			
No (779)	741 (95.1%)	38 (4.9%)	< 0.001**
Yes (167)	134 (80.2%)	33 (19.8%)	
**Low birth weight of infant**			
No (855)	786 (91.9%)	69 (8.1%)	< 0.056*
Yes (91)	89 (97.8%)	2 (2.2%)	

Data provided as *n* (%), percentages refer to column values (values in columns add up to 100); *p*-value were obtained from *Fisher’s exact test, **Pearson’s chi-square test; cut-off value for depressive symptoms was ≥ 12

^a^relatively advantaged—brahmin and Chettri

^b^relatively disadvantaged—janjati, dalit, madeshi, and muslim

### Infant neurodevelopmental delay and population characteristics

Among the infants, the IYCD development score at the 10th, 50th, and 95th percentiles were 44, 53, and 59 respectively (Additional file 1: Table S2). In the population, 7.5% of the infants had a total IYCD score ≤ 44 and were classified as having neurodevelopmental delay. There was a statistical difference in infant neurodevelopment delayed among mothers stratified by social group (*p* < 0.001), maternal education (*p* < 0.001), infant sex (*p* < 0.05), and prematurity (*p* < 0.001). Infants born to mothers with persistent depressive symptoms had a higher proportion of neurodevelopmental delay than infants born to women without persistent depressive symptoms (47.9% vs 4.6%, *p* < 0.001) (Table 2).

**Table 2:** Distribution of the co-variates for delayed infant neuro-development at 6 months using IYCD (*n* = 1253)

	**Delayed development at 6-month**		
	**No, *n* (%) 1159 (92.5%)**	**Yes, *n* (%) 94 (7.5%)**	***p*-value**
**Ethnicity (social groups)**			
Relatively advantaged^a^ (423)	409 (96.7%)	14 (3.3%)	** < 0.001**
Relatively disadvantaged^b^ (830)	750 (90.4%)	80 (9.6%)	
**Maternal education**			
Educated (1050)	996 (94.9%)	54 (5.1%)	** < 0.001**
Uneducated (135)	100 (74.1%)	35 (25.9%)	
**Infant’s sex**			
Boys (724)	498 (94.3%)	30 (5.7%)	0.052
Girl (528)	660 (91.2%)	64 (8.8%)	
**Low birth weight of infant**			
No (968)	894 (92.4%)	74 (7.6%)	0.468
Yes (285)	265 (93.0%)	20 (7.0%)	
**Preterm birth**			
No (1210)	984 (94.1%)	62 (5.9%)	** < 0.001**
Yes (43)	175 (84.5%)	32 (15.5%)	
**Depressive symptom at 7**			
Yes (127)	87 (68.5%)	40 (31.5%)	** < 0.001**
No (1126)	1072 (95.2%)	54 (4.8%)	
**Depressive symptom at 45**			
Yes (111)	69 (62.2%)	42 (37.8%)	** < 0.001**
No (850)	812 (95.5%)	38 (4.5%)	
**Depressive symptom at 90 days**			
Yes (115)	79 (68.7%)	36 (31.3%)	** < 0.001**
No (1086)	1030 (94.8%)	56 (5.2%)	
**Persistent symptom**			
Yes (71)	37 (52.1%)	34 (47.9%)	** < 0.001**
No (875)	835 (95.4%)	40 (4.6%)	

Data provided as *n* (%), percentages refer to column values (values in columns add up to 100%), level of significance = 0.05; *p*-value were obtained from *χ*^2^ test. Cut-off value for developmental delay is total IYCD score ≤ 44

^a^relatively advantaged—brahmin and Chettri

^b^relatively disadvantaged—janjati, dalit, madeshi, and muslim

### Statistical model using generalized linear regression

After adjustment for sociodemographic (social group and maternal education) and birth characteristics (infant’s sex and prematurity), infants whose mothers had depressive symptoms at 7 days had a 4.19-fold increased risk of neurodevelopmental delay compared with infants whose mothers did not have depressive symptoms (aRR, 4.19; 95% CI; 2.79, 6.30; *p* < 0.001). In the adjusted model, infants whose mothers had depressive symptoms at 90 days had a 3.32-fold increased risk of neurodevelopmental delay (aRR, 3.32; 95% CI; 2.15, 5.13; *p* < 0.001). In the adjusted model, infants whose mothers had persistent depressive symptoms had a 5.21-fold increased risk of neurodevelopmental delay compared with infants whose mothers did not have persistent depressive symptoms (aRR, 5.21; 95% CI, 3.17, 8.55; *p* < 0.001) (Additional file 1: Table S3).

### Statistical model using GLMM with the hospital as random intercept

In this model, with adjustment for sociodemographic and birth characteristics, with each increase in EPDS score at day 7, the IYCD score decreased by 0.13 (*p* = 0.005). With each increase in depressive score at all three time points (7, 45, and 90 days), the IYCD score decreased by − 0.19 (*β* coefficient =  − 0.19; *p* = 0.019) (Table 3).

**Table 3:** Mixed effects linear regression model with hospital as a random intercept on the postpartum depressive score at different time points on IYCD score

Characteristics	Beta coefficient^a^ (95% CI)	*p*-value
EPDS 7 day	− 0.13 (− 0.22, − 0.04)	0.005
EPDS 45 day	− 0.07 (− 0.17, 0.03)	0.14
EPDS 90 day	− 0.05 (− 0.16, 0.06)	0.4
EPDS at all three points	− 0.19 (− 0.36, − 0.03)	0.019

^a^Adjusted for ethnicity, maternal education, infant’s sex, and pre-term birth

### Statistical model using linear regression for individual neurodevelopmental domains

There was a difference in the mean IYCD score between infants born to mothers with persistent depressive symptoms and those born to non-depressive mothers (40.7 vs 51.9; *p* < 0.001). The mean score and adjusted regression score in the motor domain were significant when a mother had persistent depression compared to no depression (12.7 vs 15.2; *β* coefficient =  − 1.92; *p* < 0.001). The mean score and adjusted regression scores in the language domain were also significant between the two groups (6.4 vs 8.5; *β* coefficient =  − 1.80; *p* < 0.001). The mean score and adjusted regression score in the socio-emotional domain were also significant between the groups (15.4 vs 17.7; *β* coefficient =  − 3.96; *p* < 0.001). Mean score and adjusted regression score in the general behavior domain were significant when a mother had persistent depression vs no depression (5.9 vs 10.4; *β* coefficient =  − 3.96; *p* < 0.001) (Table 4 and Fig. 2).

**Table 4:** Linear regression analysis of the association between persistent maternal postnatal depression and infant neurodevelopment (individual domain) (*n* = 946)

	Persistent depression		Regression score			
	No (875)	Yes (71)				
	Mean score (SD)	Mean score (SD)	*β* coefficient, 95% CI	*p*-value	Adjusted *β* coefficient^a^, 95% CI	*p*-value
	51.9 (5.7)	40.7 (10.9)	− 11.22 (− 12.72, − 9.72)	< .001	− 9.72 (− 11.32, − 8.12)	< .001
Motor domain	15.2 (2.0)	12.7 (3.6)	− 2.37 (− 2.89, − 184)	< .001	− 1.92 (− 2.49, − 136)	< .001
language domain	8.5 (2.2)	6.4 (2.7)	− 2.15 (− 2.68, − 1.63)	< .001	− 1.80 (− 2.37, − 1.24)	< .001
Social emotional	17.7 (1.3)	15.4 (2.6)	− 2.21 (− 2.53, − 1.88)	< .001	− 2.03 (− 2.36, − 1.69)	< .001
General behavior	10.4 (2.9)	5.9 (3.3)	− 4.49 (− 5.18, − 3.81)	< .001	− 3.96 (− 4.70, − 3.23)	< .001

^a^Adjusted for ethnicity, maternal education, infant’s sex and pre-term birth

## Discussion

In this study, the prevalence of postpartum depressive symptoms persisted at 7, 45, and 90 days with similar prevalence rates, indicating that maternal stress remains high during the postpartum period. In the study cohort, one in nine infants had neurodevelopmental delay at 6 months, and the risk increased fivefold for infants born to mothers with persistent depressive symptoms up to 90 days. Due to the persistence of maternal depressive symptoms, the infants were delayed in all four functional domains, but more so in the general behavioral and social-emotional domains. Delayed infant neurodevelopment was higher among women who were from socially disadvantaged populations, had no maternal education, and were born preterm.

As the study result shows, the early infancy is a time of major transition in terms of physical and social maturation, which is influenced by the presence of maternal depression [29]. Persistent maternal depression may affect breastfeeding behavior and hinder infant stimulation and mother–child attachment, in line with our study, where the infant’s social-emotional domain and general behavior among those born to mothers with persistent depression were affected; a study in the United States reported that exposure to maternal depression was associated with difficulties in the development of infant’s social competence and emotional maturity [30]. Children exposed to maternal depression before the age of 5 had a higher risk of developmental vulnerability at school entry than children not exposed to maternal depression before the age of 5. A study in a Finnish cohort of 270 mother–child pairs showed that maternal postnatal depressive symptoms predicted low social competence in children. The children of mothers with depressive symptoms had lower problem-solving and externalizing scores than those of mothers without depression [31].

Similar to our findings of poor child development and behavioral problems in children born of mothers with postpartum depression, cohort studies in LMICs have reported similar results. A study in South Africa showed that children whose mothers had postpartum depression were more likely to have mental health problems later in life [32]. The study also showed that children from poor social groups had more social-emotional difficulties than those from relatively better social groups, which was also reported in our study. A study in Ethiopia showed that caretakers’ mental health had negative effects on personal-social, fine and gross motor, and language development [33]. The study also showed the difference in neurodevelopmental outcomes according to the sex of the infant as shown in our study. A country neighboring Nepal with similar study settings, India, has shown delayed neurodevelopment in infants whose mothers had postpartum depression [34]. This study in India also showed, as highlighted in our study, that prematurity and birth weight affect infant neurodevelopment, highlighting fetal growth as an important covariate for later development.

In settings such as Nepal, the early screening for postpartum depression has not been recommended in postpartum clinical care, resulting in early detection and management of postpartum depressive symptoms not being routine care [35]. Similarly, the system for detection and management of children with developmental delay is still under development in countries like Nepal, which mainly requires close interaction between the health and education sectors [36]. The World Health Organization recognizes maternal depression as a key health issue and recommends countries for early screening, referral, and management as part of routine postpartum care [37]. There is also a need to assess how improving the management of maternal depression reduces the risk of delayed early childhood development.

## Methodological considerations

The maintenance of a cohort of mother-infant pairs who were observed at birth during the COVID-19 pandemic is one of the major strengths of the study. The enrolled mother-infants came from diverse populations representing different hospitals and community settings, which helped to generalize the result. To our knowledge, this is one of the largest cohort studies assessing persistent maternal depression in Southeast Asia, which contributes to the generalizability of the findings. The study did not collect data on breastfeeding status at the time of IYCD assessment at 6 months, which was a major limitation, as breastfeeding, especially exclusive breastfeeding, is a meditator to maternal postpartum depression and infant neurodevelopmental status. A major limitation is that we did not assess maternal antepartum depression, which is a strong correlate of postpartum depression and has also been associated with neurodevelopmental delays [38]. Third, the IYCD is a mother-reported measure of infant neurodevelopment function and has a lower sensitivity than developmental assessment conducted using structured tools such as the Malawi Development Assessment Tool (MDAT) [39]. Finally, attrition from 2022 participants enrolled to 1253 followed up at 6 months may have introduced to sampling bias. However, when we compared the population characteristics between those lost to follow-up and those who were followed up, there was no difference in maternal ethnicity, maternal age, birth complications, and prematurity, but there was difference in infant sex (Additional file 1: Table S4).

## Conclusions

The burden of persistent maternal depression and its interlinkage with delayed infant neurodevelopment will have an unprecedented societal public health impact. The vicious cycle between poor maternal mental health and poor child developmental outcomes will be detrimental to the family and society. There is a need to further develop interventions to improve both maternal mental health and infant development through a common clinical setting so that mothers attending postpartum follow-up are screened for depressive symptoms, while infants are screened for functional neurodevelopment.

### Supplementary Information

Additional file 1. 

## Data Availability

The dataset generated and analyzed is available and provided on request.

## Abbreviations

aRR: Adjusted risk ratio
CI: Confidence interval
EPDS: Edinburgh Postnatal Depression Scale
GLM: Generalized linear modeling
IYCD: Infant and Young Child Develop Tool
LMIC: Low- and middle-income countries
MDAT: Malawi Development Assessment Tool
SDG: Sustainable development goal
