# Supplementary material for: A longitudinal multi-centric cohort study assessing infant neurodevelopment delay among women with persistent postpartum depression in Nepal
Source: BMC Med. 2024 Jul 8;22:284. doi: 10.1186/s12916-024-03501-0 (PMC11229279; doi:10.1186/s12916-024-03501-0)
Supplement: Supplementary file 1 — Additional file 1. [file 12916_2024_3501_MOESM1_ESM.docx]

Additional file 1. A longitudinal multi-centric cohort study assessing infant neurodevelopment delay among women with persistent postpartum depression in Nepal

Ashish KC^1^, Jaya Chandna^2^, Ankit Acharya^3^, Rejina Gurung^4^, Carin Andrew^4^, Alkistis Skalkidou^4^

Contents

[**Additional file 1: Table S1.** EPDS score 12 or more at three different time points 2](#_Toc169292665)

[**Additional file 1: Table S2:** IYCD development score at 6 months 3](#_Toc169292666)

[**Additional file 1: Table S3:** Generalized Linear Model (GLM) regression on postpartum depression on different time points with delayed development* among 946 women-infant pair 4](#_Toc169292667)

[**Additional file 1: Table S4.** Comparison of the socio-demographic and obstetric characteristics among women who declined to follow up and who were followed up 5](#_Toc169292668)

# **Additional file 1: Table S1.** EPDS score 12 or more at three different time points

|  | 12 or more n(%) | 11 or less n(%) |
| --- | --- | --- |
| EPDS score ≥ 12 at 7 days (1253) | 127 (10.1%) | 1126 (89.9%) |
| EPDS score ≥ 12 at 45 days (961)* | 111 (11.6%) | 850 (88.4%) |
| EPDS score ≥ 12 at 90 days (1201)** | 115 (9.6%) | 1086 (90.4%) |
| EPDS score persistently ≥ 12 (946)*** | 71 (7.5%) | 875 (92.5%) |

Lost to follow up *45 days =292, **90 days=292, *** any of three time points=307

# **Additional file 1: Table S2:** IYCD development score at 6 months

| **Centile** | **5^th^** | **10^th^** | **25^th^** | **50^th^** | **75^th^** | **90^th^** | **95^th^** |
| --- | --- | --- | --- | --- | --- | --- | --- |
| **IYCD score** | 37 | 44 | 49 | 53 | 55 | 57 | 59 |
| Gross and fine Motor | 10 | 12 | 14 | 15 | 16 | 17 | 18 |
| Language and cognitive | 4 | 5 | 7 | 9 | 10 | 11 | 11 |
| Social and emotional | 14 | 17 | 18 | 18 | 18 | 18 | 18 |
| General behavior | 5 | 6 | 8 | 11 | 13 | 13 | 14 |

# **Additional file 1: Table S3:** Generalized Linear Model (GLM) regression on postpartum depression on different time points with delayed development* among 946 women-infant pair

|  | **Standard Error (SE)** | **aRR (95% CI)**** | **p-value** |
| --- | --- | --- | --- |
| **EPDS at 7 days** |  |  |  |
| **Seven-day symptom (EPDS score ≥ 12)** | **0.870** | **4.19 (2.79, 6.30)** | **<0.001** |
| Ethnicity (advantaged group reference) | 0.427 | 1.47 (0.83, 2.60) | 0.186 |
| Maternal education (education group reference) | 0.529 | 2.63 (1.77, 3.90) | <0.001 |
| Infant sex (reference female) | 0.228 | 1.23 (0.85, 1.77) | 0.273 |
| Preterm (term reference) | 0.317 | 1.73 (1.20, 2.47) | 0.003 |
| **EPDS at 45 days** |  |  |  |
| **Forty five-day symptom (EPDS score ≥ 12)** | **1.15** | **4.91 (3.10, 7.76)** | **<0.001** |
| Ethnicity (advantaged group reference) | 0.99 | 2.49 (1.14, 5.44) | 0.022 |
| Maternal education (education group reference) | 0.50 | 2.38 (1.58, 3.58) | <0.001 |
| Infant sex (reference female) | 0.18 | 1.08 (0.77, 1.51) | 0.675 |
| Preterm (term reference) | 0.28 | 1.54 (1.08, 2.20) | 0.017 |
| **EPDS at 90 days** |  |  |  |
| **Ninety-day symptom (EPDS score ≥ 12)** | **0.73** | **3.32 (2.15, 5.13)** | **<0.001** |
| Ethnicity (advantaged group reference) | 0.52 | 1.72 (0.95, 3.12) | 0.075 |
| Maternal education (education group reference) | 0.58 | 2.63 (1.71, 4.05) | <0.001 |
| Infant sex (reference female) | 0.25 | 1.26 (0.85, 1.87) | 0.241 |
| Preterm (term reference) | 0.32 | 1.59 (1.07, 2.36) | 0.022 |
| **Persistent depression** |  |  |  |
| **Persistent depression (EPDS score ≥ 12)** | **1.32** | **5.21 (3.17, 8.55)** | **<0.001** |
| Ethnicity (advantaged group reference) | 0.29 | 1.40 (0.94, 2.09) | 0.102 |
| Maternal education (education group reference) | 0.91 | 2.30 (1.06, 5.01) | 0.035 |
| Infant sex (reference female) | 0.50 | 2.18 (1.39, 3.41) | 0.001 |
| Preterm (term reference) | 0.21 | 1.11 (0.77, 1.60) | 0.59 |

**delayed neurodevelopment = IYCD total score≤44;* ***adjusted for Ethnicity, maternal education, infant’s sex and pre-term birth*

# **Additional file 1: Table S4.** Comparison of the socio-demographic and obstetric characteristics among women who declined to follow up and who were followed up

|  | Not included (769) | Followed up (1253) | Pearson's chi-square test (p-value) |
| --- | --- | --- | --- |
| **Ethnicity** |  |  | 0.29 |
| Dalit (237) | 97 (12.6%) | 140 (11.2%) |  |
| Janajati (594) | 243 (31.6%) | 351 (28.0%) |  |
| Madhesi (432) | 163 (21.2%) | 269 (21.5%) |  |
| Muslim (85) | 32 (4.2%) | 53 (4.2%) |  |
| Brahmin/Chhetri (649) | 226 (29.4%) | 423 (33.8%) |  |
| Others (25) | 8 (1.0%) | 17 (1.4%) |  |
| **Maternal age in years** |  |  | 0.439 |
| <19 (80) | 37 (4.8%) | 43 (3.4%) |  |
| 19-24 (1017) | 389 (50.6%) | 628 (50.1%) |  |
| 25-29 (639) | 239 (31.1%) | 400 (31.9%) |  |
| >30 (286) | 104 (13.5%) | 182 (14.5%) |  |
| **Complication during admission** |  |  | 0.509 |
| No (1787) | 675 (87.8%) | 1112 (88.7%) |  |
| Yes (235) | 94 (12.2%) | 141 (11.3%) |  |
| **Complication during birth** |  |  | 0.645 |
| No (1817) | 688 (89.5%) | 1129 (90.1%) |  |
| Yes (205) | 81 (10.5%) | 124 (9.9%) |  |
| **Mode of delivery** |  |  | 0.298 |
| Sponatenous Vaginal Delivery (1457) | 566 (73.6%) | 891 (71.1%) |  |
| Instrumental Delivery (60) | 25 (3.3%) | 35 (2.8%) |  |
| Cesarean delivery (505) | 178 (23.1%) | 327 (26.1%) |  |
| **Preterm birth** |  |  |  |
| Term (1676) | 630 (81.9%) | 1046 (83.5%) | 0.367 |
| Preterm (346) | 139 (18.1%) | 207 (16.5%) |  |
| **Infant sex** |  |  | <0.001 |
| Boy (920) | 392 (51.0%) | 528 (42.2%) |  |
| Girl (1101) | 377 (49.0%) | 724 (57.8%) |  |
